# *Klebsiella pneumoniae* NdpA suppresses ERK pathway-mediated host early inflammatory responses and is degraded through the ubiquitin-proteasome pathway

**DOI:** 10.1007/s13238-016-0341-y

**Published:** 2016-11-14

**Authors:** Guanghua Xu, Jing Wang, Cui Hua Liu

**Affiliations:** 1 CAS Key Laboratory of Pathogenic Microbiology and Immunology, Institute of Microbiology, Chinese Academy of Sciences, Beijing, 100101 China; 2 School of Life Sciences, Anhui University, Hefei, 230601 China; 3 Savaid Medical School, University of Chinese Academy of Sciences, Beijing, 101408 China

**Dear Editor**,

*Klebsiella pneumoniae* (KP) is an important opportunistic pathogen causing community-acquired and nosocomial infections. When the host is immunocompromised, the pathogen would infect the host and cause diseases, such as pneumoniae, sepsis, liver abscess, meningitis, urinary tract inflammation and wound infection (Karaiskos et al., 2016; Park et al., 2015). The phenomenon that *K. pneumoniae* has a preference to infect immunocompromised populations, especially seniors suggests that the outcomes of *K. pneumoniae* infection depend on the pathogen-host interactions, but up to now, the molecular mechanisms underlying *K. pneumoniae*-host interactions remain largely unknown.

Previous studies reported that *K. pneumoniae* suppresses inflammatory cytokine production during early period of infection (Lawlor et al., 2006), and this bacteria can block the activation of inflammatory responses by antagonizing NF-κB and MAPK signaling pathways (Frank et al., 2013; Regueiro et al., 2011). We discovered that the *K. pneumoniae* nucleoid-associated protein (NdpA) is highly conserved among gram-negative bacteria. Transient expression of NdpA in human embryonic kidney HEK239T cells inhibited the Elk activation induced by RasV12, as well as V-Raf (constitutive active Raf) and MEK1-ED (constitutive active MEK1), respectively (Fig. 1A–C). *K. pneumoniae* NdpA also promoted tumor necrosis factor (TNF) α-stimulated NF-κB activation (Fig. S1A), and had little, if any, inhibitory effect on JNK and p38 signaling pathways (Fig. S1B). Given the lack of inflammation at the early stage of *K. pneumoniae* infection (Lawlor et al., 2006), we thus focused on the elucidation of the suppressive effects of *K. pneumoniae* NdpA on ERK signaling pathway-mediated host early inflammatory responses. Because many pathogenic bacteria have secretion systems to inject their virulence factors into host cells to interfere their functions. Thus, we sought to examine whether *K. pneumoniae* NdpA could be secreted into host cells during infection. Immunoblot analysis showed that NdpA entered into the cytosol of the human alveolar epithelial cells A549 during *K. pneumoniae* infection (Fig. S2A). In addition, we found that the phosphorylation of ERK1/2 activated by MEK1-ED was largely reduced by NdpA (Fig. 1D). Consistently, NdpA also abolished extracellular stimuli epidermal growth factor (EGF)-activated ERK1/2 phosphorylation (Fig. 1E). To further confirm the role of NdpA in the suppression of ERK signaling during *K. pneumoniae* infection, we tried to knockout the gene encoding NdpA with several methods available, but after many attempts we failed to obtain the expected mutant strain. We thus adopted the alternative strategy to investigate the host immune-regulatory function of NdpA by overexpressing it in *K. pneumoniae*. We found that overexpression of NdpA in *K. pneumoniae* and *E. coli* resulted in down-regulation of phospho-ERK1/2 (p-ERK1/2) in A549 cells during *K. pneumoniae* infection (Figs. 1F and S2B).

**Figure 1:**
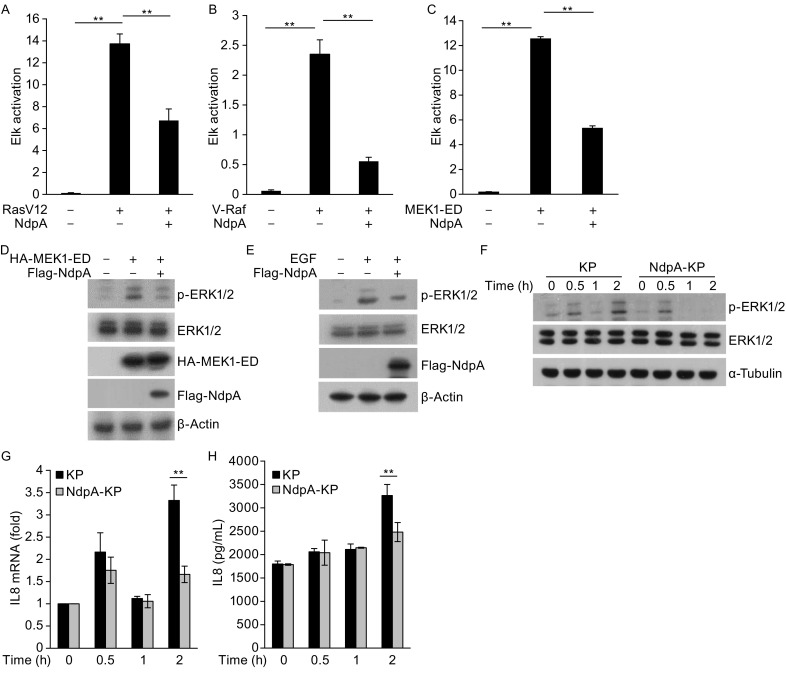
***K. pneumoniae*** **NdpA inhibits ERK1/2 signaling pathway activation**. (A–C) Dual-luciferase assays of RasV12 (A)-, V-Raf (B)-, and MEK1-ED (C)- activated ERK1/2 signaling in the absence or presence of NdpA. Data are representative of at least three independent experiments (mean and SEM). *P < 0.05, **P < 0.01 (two-tailed unpaired *t*-test). (D–F) The effects of NdpA on ERK1/2 phosphorylation as assessed by immunoblotting. HEK293T cells expressing MEK1-ED without or with NdpA (D); HEK293T cells transfected with empty vector or vector encoding flag-tagged NdpA were treated with 100 ng/mL of EGF for 15 min (E); A549 cells were infected with WT or NdpA-overexpressing *K. pneumoniae* (NdpA-KP) for indicated time (F). (G) Quantitative real-time PCR analysis of *Il8* mRNA in A549 cells infected with KP or NdpA-KP for indicated time. (H) ELISA analysis of IL8 in the supernatants of A549 cells infected with KP or NdpA-KP for indicated time, respectively

With a central role in recruiting and infiltrating neutrophils into inflammatory sites, IL8 is known as a main inflammatory molecule involved in host defense against *K. pneumoniae* infection (Harada et al., 1994). We thus next explored whether NdpA regulates the expression of IL8 in human alveolar epithelial cells during *K. pneumoniae* infection. The data from quantitative real-time PCR showed that compared with the wild-type (WT) *K. pneumoniae* strain, the NdpA-overexpressing *K. pneumoniae* strain significantly down-regulated the mRNA of IL8 in A549 cells (Fig. 1G). Accordingly, the secretion of IL8 was apparently attenuated by the overexpression of NdpA in *K. pneumoniae* as analyzed by enzyme-linked immunosorbent assay (ELISA) (Fig. 1H). To determine whether the inhibitory effects of NdpA on IL8 production is dependent on ERK signaling pathway, we pretreated A549 cells with U0126, a specific inhibitor of ERK pathway, before the infection assay, and we found that the WT *K. pneumoniae* strain and the NdpA-overexpressing *K. pneumoniae* strain showed similar amount of IL8 production during infection of A549 cells (Fig. S3A and S3B).

We further sought to investigate the underlying mechanisms by which NdpA suppresses host inflammatory responses. Based on the observation that NdpA inhibited ERK1/2 phosphorylation stimulated by MEK1-ED, the activator of ERK pathway next to ERKs (Fig. 1D), MEK1 and ERKs were chosen to verify their interactions with NdpA. HEK293T cells expressing Flag-tagged ERK2 or Myc-tagged MEK1 were used for co-immunoprecipitation analysis with His-tagged NdpA purified from *E. coli* BL21, and the data indicated that NdpA could specifically bind to ERK2, but not MEK1 (Fig. S4A). Furthermore, we found that overexpression of NdpA in HEK293T cells completely abolished the binding of MEK1 to ERK2 (Fig. S4B).

Through protein sequence alignment analysis, we found that NdpA harbors a canonical D motif (15-KRDEQNLEL-23) for ERKs docking. The D motif contains the consensus sequence (K/R)_1-2_-(X)_2-6_-Ø_A_-X-Ø_B_ (Ø_A_ and Ø_B_ are hydrophobic residues) (Supplementary Fig. 4C) (Zhou et al., 2006; Zhu et al., 2007). To identify which site plays the most critical role in the binding to ERK1/2, four mutants of NdpA were constructed, including NdpA (K15A), NdpA (R16A), NdpA (L21E) and NdpA (L23E). Through dual-luciferase assay, we found that NdpA (L21E) evidently impaired the inhibitory effects of NdpA on ERK pathway (Fig. S4D). His-tag pull-down analysis further confirmed that NdpA could directly bind to ERK2 in vitro, but the NdpA (L21E) mutant had little binding affinity (Supplementary Fig. 4E). Consistently, NdpA (L21E) lost the ability to block the interactions between ERK2 with MEK1 (Fig. S4F). Unlike WT NdpA, NdpA (L21E) could not efficiently inhibit MEK1-ED-stimulated ERK1/2 phosphorylation in HEK293T (Supplementary Fig. 5A), and also failed to inhibit ERK1/2 phosphorylation in A549 cells during *K. pneumoniae* infection (Supplementary Fig. 5B). Consistently, quantitative real-time PCR and ELISA analysis also showed that NdpA (L21E) couldn’t effectively downregulate IL8 expression in A549 cells during *K. pneumoniae* infection (Fig. S5C and S5D). Together, these data indicate that NdpA inhibited ERK signaling pathway-mediated inflammation in a D motif-dependent manner.

Since the suppressive effects of *K. pneumoniae* are usually attenuated at about 4 hours post infection of host cells, we thus further questioned whether the host could counteract the regulatory effects of NdpA during the course of *K. pneumoniae* infection by regulating the protein stability of NdpA. Interesting, by online bioinformatics analysis (http://smart.embl-heidelberg.de/), we identified two tandem ubiquitin (Ub)-interacting Motifs (UIMs) in *K. pneumoniae* NdpA. The UIM motif has been reported to occur more frequently in eukaryotic cells instead of prokaryotic cells and it is specific for interacting with free Ub, poly-ubiquitin chains, as well as ubiquitin-conjugated proteins (Polo et al., 2003). Therefore, we hypothesized that NdpA might be degraded by the host through an ubiquitin-dependent pathway. When the vectors encoding NdpA and ubiquitin were co-transfected in HEK293T cells for co-immunoprecipitation analysis, we observed that NdpA was polyubiquitinated in vivo (Fig. 2A). Furthermore, overexpression of ubiquitin in HEK293T cells caused the reduction of NdpA protein (Fig. 2B). To identify whether the two UIMs play a role in the polyubiquitination of NdpA, a few truncated mutants of UIM were constructed as indicated in Fig. S6A, co-immunoprecipitation analysis data indicated that the two UIMs are dispensable for the polyubiquitination of NdpA (Fig. S6B), we thus surmised that the UIM motif in NdpA might participate in other currently unknown regulatory functions in hosts.

**Figure 2:**
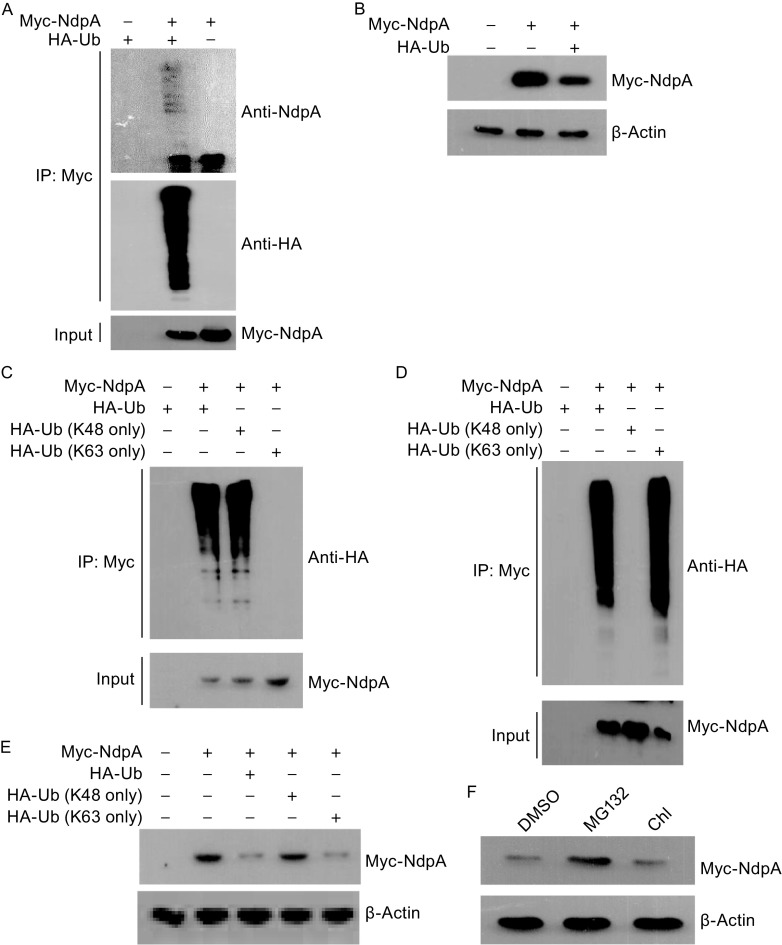
***K. pneumoniae*** **NdpA is degraded through the ubiquitin-proteasome pathway in host cells**. (A) Ubiquitination assay of NdpA in vivo. HEK293T cells were transfected with indicated plasmids, 24 h later, Myc-NdpA was immunuoprecipated with the gel-conjugated Myc antibody and immunoblotted with anti-HA and anti-NdpA antibodies. (B) Immunoblotting analysis of Myc-NdpA in the absence or presence of HA-Ub in HEK293T cells. (C) HEK293T cells expressing Myc-NdpA together with HA-Ub or Lys 48-linked polyubiquitylation (K48 only) or Lys 63-linked polyubiquitylation (K63 only) were subjected to the gel-conjugated Myc antibody and immunoblotted with HA antibody. (D) HEK293T cells expressing Myc-NdpA together with HA-Ub or HA-Ub (K48R) or HA-Ub (K63R) were subjected to the gel-conjugated Myc antibody and immublotted with HA antibody. (E) A549 cells transfected with indicated plasmids were detected for NdpA protein by immunoblotting. (F) A549 cells expressing Myc-NdpA were treated with DMSO or 10 μmol/L MG132 or 100 μmol/L chroloquine (Chl) for 6 h, and then NdpA protein was detected by immunoblotting

We next sought to determine the type of ubiquitin chains in NdpA, and we found that NdpA was effectively polyubiquitinated by Lys 48-linked polyubiquitin (K48 only) chains, but not the non-degradative Lys 63-linked polyubiquitin (K63 only) chains (Fig. 2C). Furthermore, the ubiquitin mutant (K48R) instead of the ubiquitin mutant (K63R) couldn’t promote NdpA polyubiquitination (Fig. 2D). Consistently, ubiquitin (K48R) lost the ability to destabilize NdpA protein, while ubiquitin (K63R) promoted degradation of NdpA as same as the WT ubiquitin (Fig. 2E). These results indicated that NdpA might be degraded through the proteasome pathway since Lys 48-linked polyubiquitination is commonly associated with the proteasome pathway-dependent protein degradation. We then found that MG132, the inhibitor of proteasome, indeed stabilized the NdpA protein (Fig. 2F). Increasing number of evidences revealed that ubiquitin could also target substrates to lysosome for degradation (Lu et al., 2014; Pankiv et al., 2007). But our data showed that the lysosome inhibitor chloroquine failed to stabilize NdpA (Fig. 2F). A previous study reported that ubiquitin could mediate ERK1/2 degradation through proteasome-dependent pathway (Lu et al., 2002), we thus examined whether NdpA affects ERK1/2 polyubiquitination and degradation. Our data showed that overexpression of NdpA had little effect on the polyubiquitiniatin of ERK2, as well as the endogenous ERK1/2 protein levels (Fig. S7A and S7B). Collectively, *K. pneumoniae* NdpA undergoes Lys 48-linked polyubiquitination-mediated proteasomal degradation in host cells.

In this study, we revealed that NdpA, a secreted effector protein of *K. pneumoniae*, specifically inhibits ERK signaling pathway, leading to the suppression of host early inflammatory responses. We found that NdpA from *K. pneumoniae* abrogates ERK pathway-mediated expression of IL8 in a D motif-dependent manner. Sequence alignment results indicate that NdpA is a highly conserved protein widely exists in gram-negative bacteria, most of them share the same or similar protein sequence of D motif with *K. pneumoniae* NdpA. Therefore, the D motif might provide selectivity for the development of novel pan-anti-gram-negative pathogen therapies.

While in immunocompromised hosts (such as the aged patients) whose innate immunity such as proteasome function is attenuated during aging process (Tonoki et al., 2009), the bacteria might gain the upper hand and cause diseases. Thus our findings help to explain, at least partially, that why *K. pneumoniae* tends to infect immunocompromised hosts such as the seniors.

## Electronic supplementary material

Below is the link to the electronic supplementary material.

Supplementary material 1 (PDF 636 kb)
