# Supplementary material for: Klebsiella pneumoniae NdpA suppresses ERK pathway-mediated host early inflammatory responses and is degraded through the ubiquitin-proteasome pathway
Source: Protein Cell. 2016 Nov 14;8(2):144–8. doi: 10.1007/s13238-016-0341-y (PMC5291776; doi:10.1007/s13238-016-0341-y)
Supplement: Supplementary file 1 — Supplementary material 1 (PDF 636 kb) [file 13238_2016_341_MOESM1_ESM.pdf]

Supplementary materials

MATERIALS AND METHODS

Bacterial strains, growth conditions and plasmids

K. pneumoniae, *E. coli* DH5α, *E. coli* BL21 (DE3) and *E. coli* ATCC25922 were grown in Luria-Bertani (LB) broth medium at 37 ℃. *K. pneumonia* and *E. coli* ATCC25922 was used for infection. *E. coli* DH5α and *E. coli* BL21 were used for amplifying plasmids and extracting proteins, respectively. The dual-luciferase reporter assay vectors (Gal4-luc, Gal4-Elk, pRL-TK, pFA-cJun and pNF-κB-luc), RacL61, RasV12, v-Raf, MEK1-ED, pcDNA6A-MEK1, pcDNA-HA-Ub, pcDNA-HA-Ub (K48R) and pcDNA-HA-Ub (K63R) were provided by Feng Shao (National Institute of Biological Sciences, Beijing, China). p3×Flag-CMV-14-ERK2 and pGEX-6p-1-ERK2 was kept in our laboratory. pcDNA-HA-Ub (K48 only) and pcDNA-HA-Ub (K63 only) were provided by Lingqiang Zhang (Beijing Institute of Radiation Medicine). The *K. pneumonia ndpa* gene was synthesized and cloned into plasmid pET-28a. *K. pneumoniae* expression plasmid pACYC177 was provided by Youjun Feng (Zhejiang University). For mammalian cell expression vectors, the PCR fragments were inserted into the vector p3×Flag-CMV14 (Flag tag) or pcDNA6A (Myc tag). Point and truncated mutants of NdpA were generated using Mut Express

Ⅱ Fast Mutagenesis Kit V2 (C214-01; Vazyme). The detailed information on strains, plasmids, and oligonucleotides used in this study are list in Supplementary Table 1.

Antibodies and reagents

The commercial antibodies used in this study include: anti-ERK1/2 (9102; CST), anti–p-ERK1/2 (9101; CST), anti-GST (TA-03; ZSGB-BIO), anti-Myc (sc-40; Santa Cruz), anti-Flag (F3165; Sigma-Aldrich), anti-α-Tubulin (T6199; Sigma-Aldrich), anti-poly (ADP-ribose) polymerase Ab (9542; CST), anti-HA (3724S; CST), and anti-βactin (A2228; SigmaAldrich). Anti-NdpA was generated by immunizing rabbit using His-NdpA. Ni-NTA Agarose was from Qiagen; Glutathione Sepharose 4B was from GE Healthcare; Mouse anti-Flag M2 affinity gel (F3165), U0126 ethanolate (U120), and epidermal growth factor (EGF) (E9644) were from Sigma-Aldrich; c--Myc Antibody agarose cojugate for IP (SC40AC) was from Santa Cruz.

Cell culture and infection

A549, HEK293T and Hela cells were grown in DMEM tissue culture medium supplemented with 10% heated-inactivated fetal bovine serum and 1% antibiotics (penicillin and streptomycin) in 10 cm cell culture dishes at 37 ℃ in a water-saturated atmosphere containing 5% CO_2. For infection, A549 cells were seeded to about 90% confluence (1.4×10^6 per well) in 6-well tissue culture plates. Before infection, cells

were serum starved for 12 to 16 h. The infection was performed at a multiplicity of infection (M.O.I) of 150 *K. pneumoniae* or 100 *E. coli* ATCC25922 per cell.

Cell transfection, immunoblotting and immunoprecipitation

HEK293T cells were transfected using (PEI) or standard calcium phosphate method. A549 or HeLa cells were transfected with Lipofectamine 2000 (Invitrogen, Carlsbad, CA) following the manufacture's instruction. For immunoblotting, HEK293T or A549 cells were seeded in 6-well plates and grown to about 70% confluence, then vectors were transfected, four to six hours after transfection, cells were fed with fresh tissue culture medium. After 24-48 h, cells were harvested and lysed with the Cell Lysis Buffer for Western and IP (P0013, Beyotime). Total cell lysates were separated by SDS-PAGE and transferred to polyvinylidenedifluoride (PVDF) membrane (Millipore). The membrane was block with 5% skimmed milk in Tris-buffered saline (TBS) for 1 h at room temperature and subsequently incubated with appropriate primary antibodies in TBS with 1% (V/V) twwen-20 (TBST) and 5% (W/V) skimmed milk at 4 ℃ overnight. Following three washes of 10 min each with TBST, the PVDF membranes were incubated with goat anti-mouse or goat anti-rabbit IgG conjugated to HRP at a dilution of 1:10000 in TBST for 1 h at room temperature. After three washes with TBST, the membrane was subjected to Immobilon Western Chemiluminescent HRP Substrate (WBKLS0500; Millipore) and exposed to X-ray film. For immunoprecipitation, transfected HEK293T cells were lysed with buffer containing

20 mM Tris (pH 7.5), 150 mM NaCl, 1% Triton X-100, and a protease inhibitor mixture (P8340; Sigma). Cell lysates were incubated with Flag M2 beads (A2220; Sigma) or anti-Myc beads (sc-40AC; Santa CruZ) at 4℃ for 4 h, followed by extensive wash with the lysis buffer. Both lysates and immunoprecipitates were examined by immunoblotting.

Dual-luciferase assay

Dual luciferase assay was performed using the Promega luciferase reporter system. HEK293T cells were seeded in 12-well plates overnight. For NF-κB pathway, HEK293T cells were co-transfected with1 μg pNF-kB-Luc and 50 ng pRL-TK in the presence or absence of 1 μg NdpA plasmid. At 24 h later, NF-κB pathway was stimulated with 20 ng/ml TNF (Invitrogen) for 6 h. For ERK pathway, HEK293T cells were co-transfected with 0.6 μg Gal4-Elk, 0.6 μg Gal4-luc, 50 ng pRL-TK and 10 ng RasV12, or 100 ng v-Raf or 100 ng constitutive active MEK1 (MEK1-ED) with or without 1 μg NdpA. For JNK and p38 pathways, HEK293T cells were co-transfected with 0.3 μg pFA-cJun, 0.9 μg Gal4-luc, 0.5 μg RacL61, and 50 ng pRLTK in the presence or absence of 1 μg NdpA plasmid.

Protein purification and His pull-down

All proteins were purified from *E. coli* BL21 (DE3). His-NdpA, His-NdpA (L21E) and GST-ERK2 were induced at 30°C overnight with 100 μM isopropyl-β-D-thiogalactopyranoside (IPTG) upon OD_{600} reached 0.6-0.8. His tagged proteins were purified by affinity chromatography using Ni-NTA Agarose, GST-ERK2 was purified by Glutathione Sepharose 4B. For His pull-down, the His fusions of proteins were immobilized onto Ni-NTA Agarose followed by incubation with prey proteins in lysis buffer containing 50 mM NaH_2PO_4 (pH 8), 300 mM NaCl, 20 mM imidazole and 1% Triton X-100, or HEK293T cell lysate supplemented with protease inhibitor mixture at 4˚C for 4 h. The beads were extensively washes four or five times. The bound proteins were analyzed by immunoblotting.

Cell fractionation

Cells were lysed on ice for 5 min with buffer containing 10 mM HEPES (pH7.9), 1.5 mM $MgCl_2$, 10 mM KCl, 0.34 M sucrose, 10% glycerol, protease inhibitors and 0.1% Triton X-100, followed by centrifugation at 13,000 g for 10 min. The supernatant was collected as cytosolic fraction. The pellet was resuspended with 1 × PBS plus 0.1% SDS and 0.1% Triton X-100, followed by centrifugation in a 15% sucrose cushion twice at 2,500 g for 15 min at 4 ℃.The supernatant was the nuclear fraction.

Quantitative PCR and enzyme-linked immunosorbent assay

A549 cells seeded into 6-well plates were infected as described above. After infection, the cells culture media were collected for ELISA, the cells were washed with 1 ×PBS and extracted total RNA using commercial kit (R1061). Subsequently the RNA was reverse transcribed into cDNA using commercially available kits follows the manufacturers' instructions. The cDNA was then analyzed by quantitative PCR with KAPASYBR FAST qPCR Kit (KAPABiosystems) on ABI 7300 system (Applied Biosystems). Data were normalized to Gapdh. For ELISA, IL8 in supernatant was determined using Human IL8 ELISA Kit (F2391-B; TSZ)

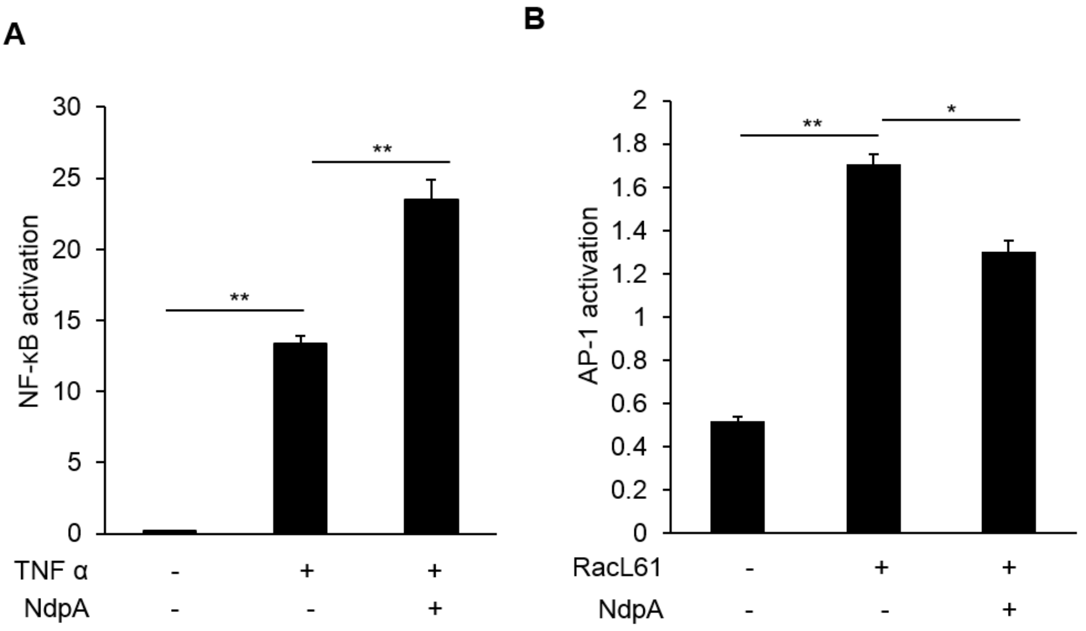

SUPPLEMENTARY FIGURE 1. The effects of *K. pneumoniae* NdpA on NF-κB and JNK/p38 signaling pathways. Dual-luciferase assay of TNFα-activated NF-κB pathway (A) and constitutively active RacL61-activated JNK and p38 pathways (B) in the absence or presence of NdpA. Data are representative of at least three independent experiments (mean and SEM). **P* < 0.05, ** *P* < 0.01 (two-tailed unpaired *t*-test).

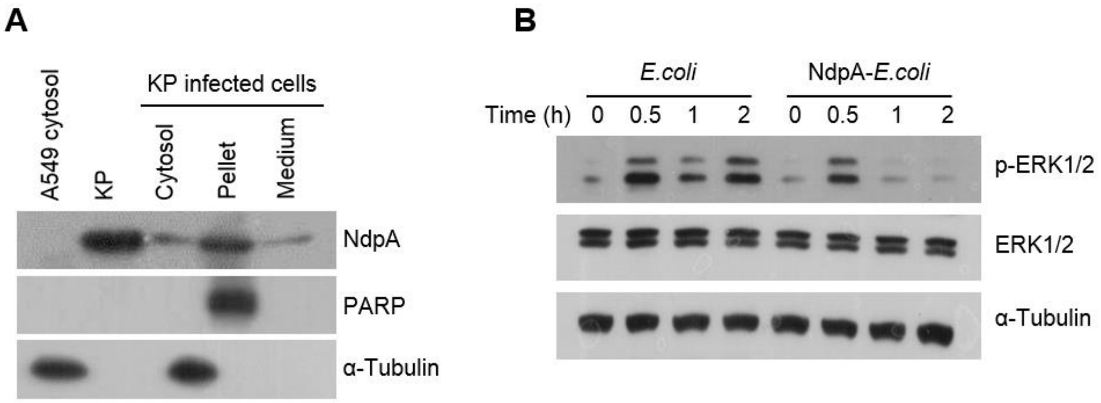

SUPPLEMENTARY FIGURE 2. The effects of NdpA on ERK 1/2 phosphorylation. (A) Cell fractionation analysis of NdpA in *K. pneumoniae* (KP)-infected A549 cells. Nuclei and bacteria were collected as pellet. α-Tubulin and poly (ADP-ribose) polymerase (PARP) were served as markers of cytosol and nucleus, respectively. (B) A549 cells were infected for indicated time with WT or NdpA-overexpressing *E.coli* ATCC25922.

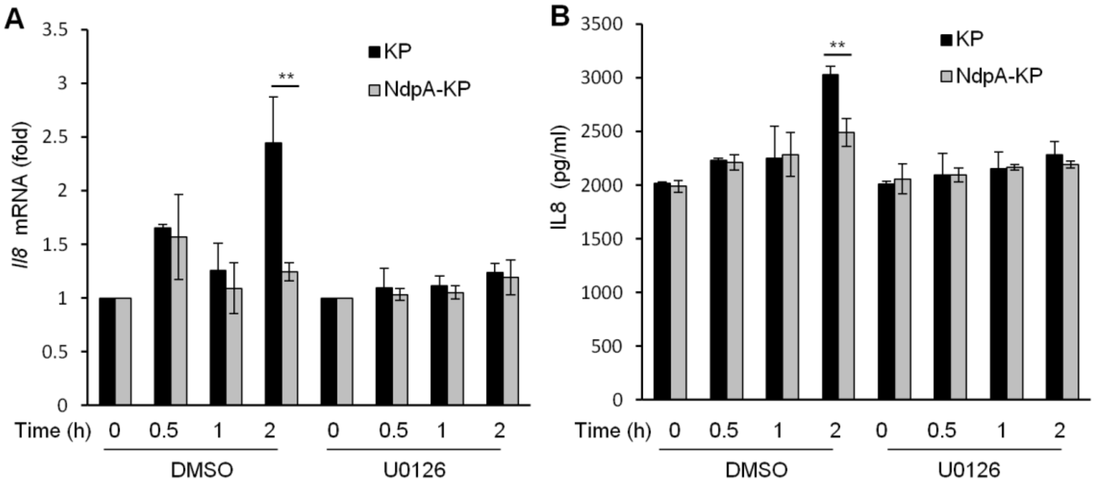

SUPPLEMENTARY FIGURE 3. NdpA downregulates IL8 production dependent on ERK signaling pathway. (A) Quantitative PCR analysis of *Il8* mRNA in A549 cells infected for indicated time with KP or NdpA-KP, respectively. Cells were treated with 10 μM of U0126 before infection. (B) ELISA analysis of IL8 in the supernatants of A549 cells infected as in (A). Data are representative of at least three independent experiments (mean and SEM). *P < 0.05, **P < 0.01 (two-tailed unpaired t-test).

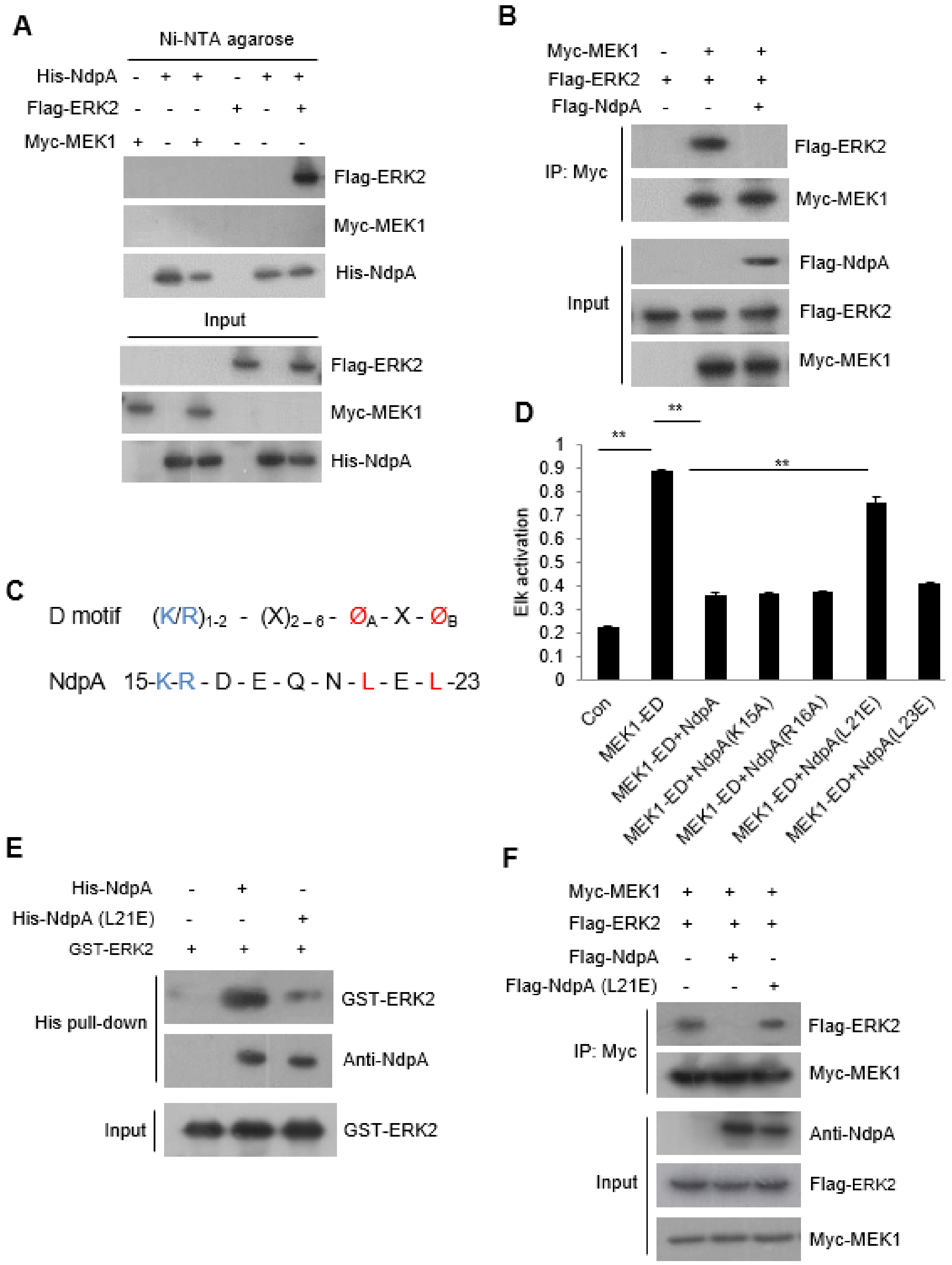

SUPPLEMENTARY FIGURE 4. NdpA binds to ERK via the D motif. (A) NdpA interacts with ERK, but not MEK1. Whole HEK293T cell lysates expressing Flag-ERK2 or Myc-MEK1 were subjected to Ni-NTA agarose with His-NdpA. (B)

NdpA interrupts the binding between MEK1 and ERK. Whole HEK293T cell lysates expressing Flag-ERK2 and Myc-MEK1 without or with Flag-NdpA incubated with flag affinity gel. (C) Schematic drawing of the D motif and NdpA. Blue, basic residues; red, hydrophobic residues; X, arbitrary residues. (D) Dual-luciferase assay of MEK1-ED-activated ERK signaling pathway in the presence of WT NdpA, NdpA (K15A), NdpA (R16A), NdpA (L21E) and NdpA (L23E), respectively. Data are representative of at least three independent experiments (mean and SEM). *P<0.05, **P<0.01 (two-tailed unpaired *t*-test). (E) NdpA, but not NdpA (L21E), bounds to ERK2 in vitro. GST-ERK2 with His-NdpA or His-NdpA (L21E) were precipitated by Ni-NTA agarose. (F) The effects of NdpA (L21E) on the interactions between MEK1 and ERK2. The whole HEK293T cell lysates expressing Flag-ERK2 and Myc-MEK1 with or without Flag-NdpA or Flag-NdpA (L21E) were co-immunoprecipitated by the gel-conjugated Myc antibody.

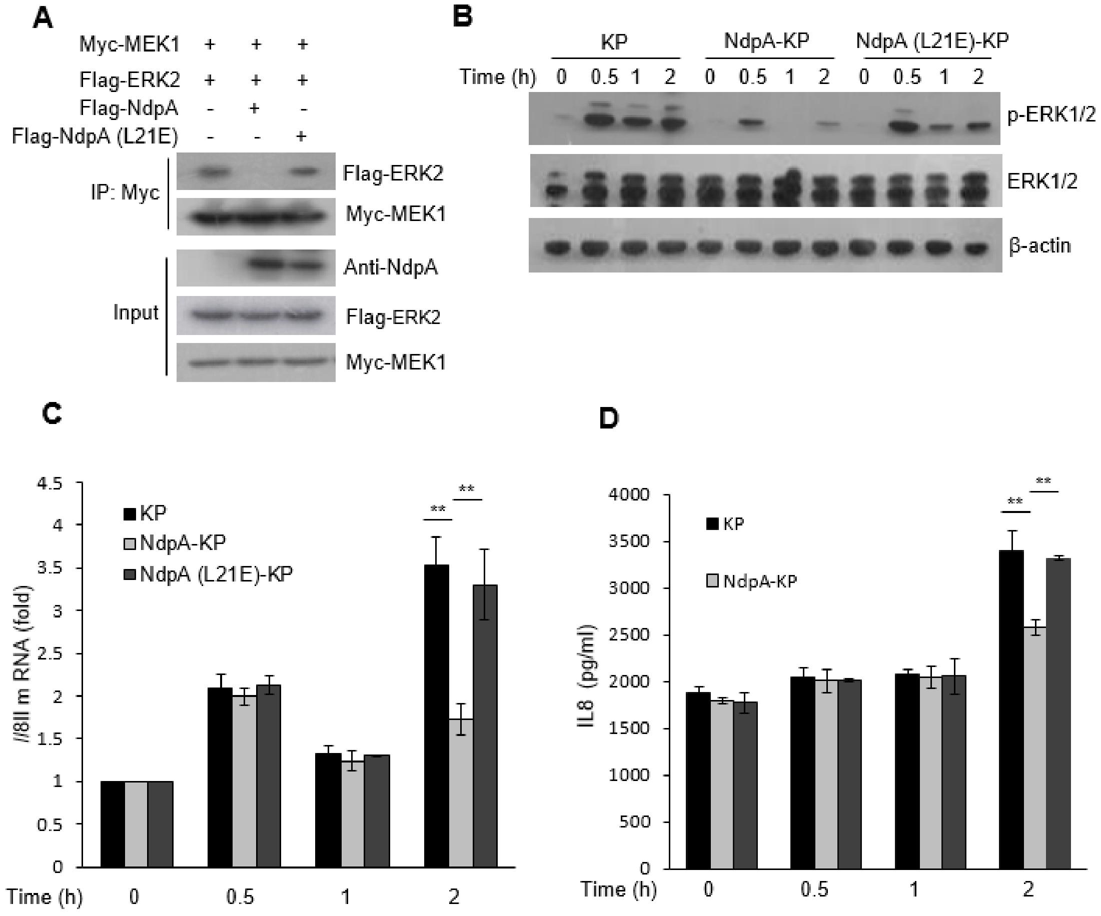

SUPPLEMENTARY FIGURE 5. D motif is essential for NdpA to suppress ERK pathway-mediated IL8 production. (A-B) Immunoblotting analysis of the phosphorylation of ERK1/2 in host cells. HEK293T cells were activated by MEK1-ED in the presence or absence of NdpA or NdpA (L21E) (A); A549 cells were infected with KP, NdpA-KP or NdpA (L21E)-KP, respectively (B). (C) Quantitative PCR analysis of *Il8* mRNA in A549 cells infected for indicated time with KP or NdpA-KP or NdpA (L21E)-KP, respectively. (D) ELISA analysis of IL8 in the supernatants of A549 cells infected for 0-2 h with KP or NdpA-KP or NdpA (L21E)-KP, respectively.

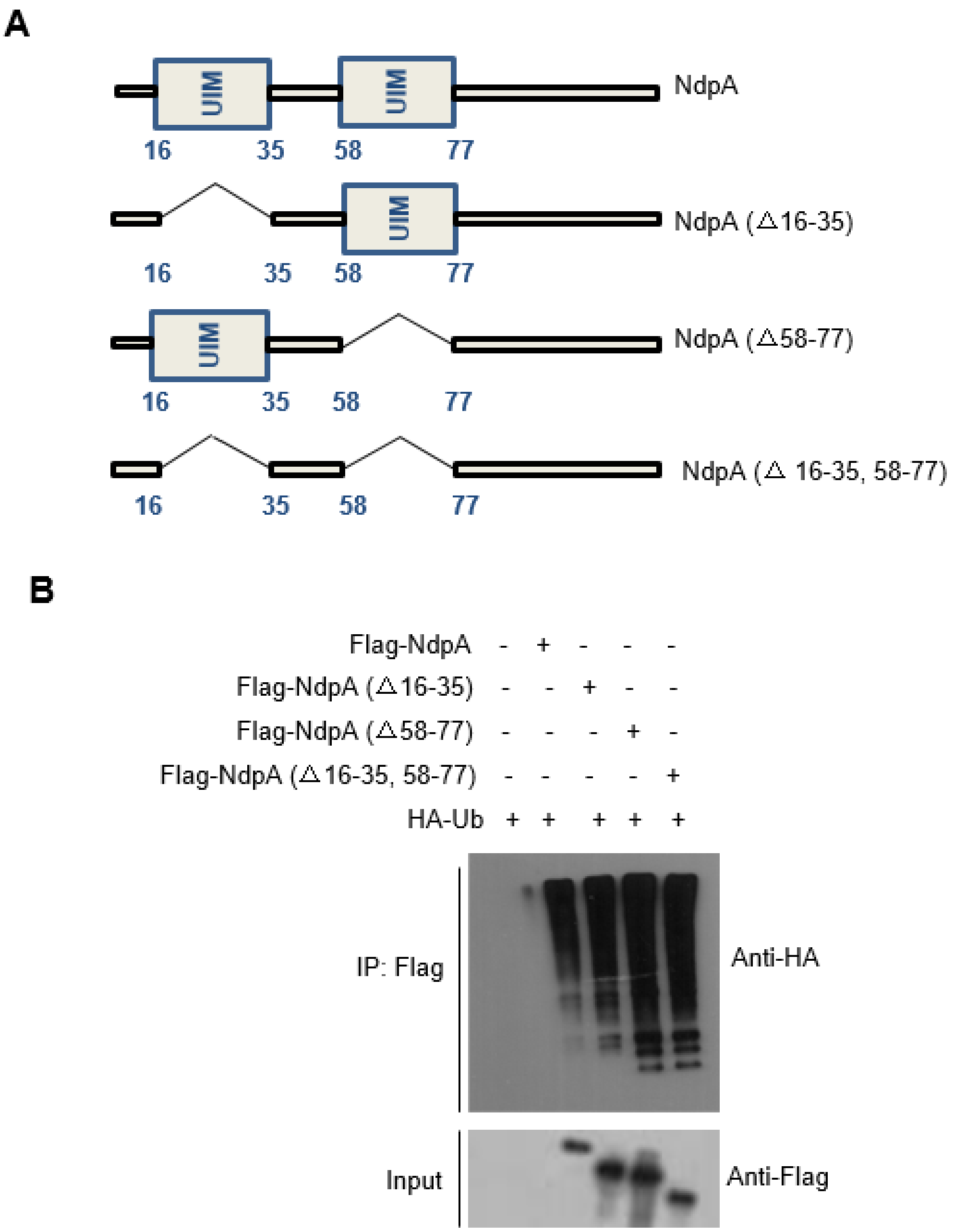

SUPPLEMENTARY FIGURE 6. The role of the UIM domain in NdpA polyubiquitination. (A) The schematic drawings of truncated UIM mutants of NdpA. (B) HEK293T cells were transfected with the indicated plasmids, Flag-tagged NdpA and its truncated mutants were immunoprecipitated (IP) with anti-Flag antibody and immunoblotted with anti-HA antibody.

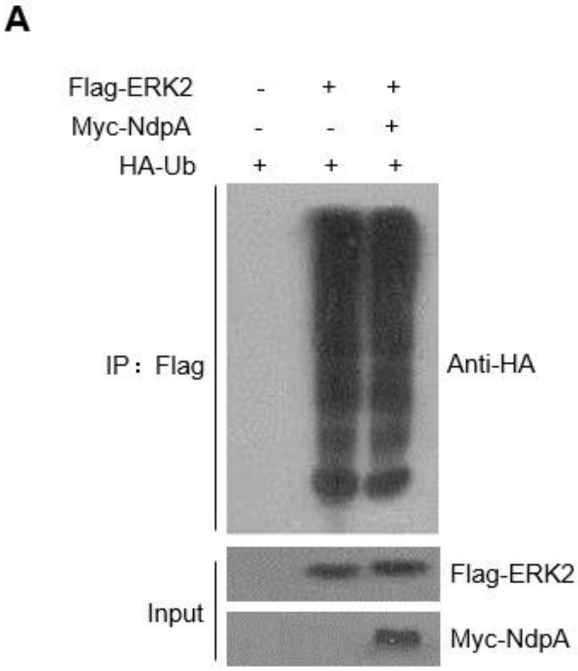

SUPPLEMENTARY FIGURE 7. The influence of NdpA on the polyubiuitination and degradation of ERK1/2. (A) HEK293T cell lysates transfected with indicated plasmids were subjected to IP with Flag antibody and immunoblotted with anti-HA antibody. (B) HEK293T cells were transfected with Myc-tagged NdpA plasmid in the absence or presence of HA-Ub plasmid. After 48h, cell lysates were used to detect ERK1/2 protein levels.

Supplementary Table 1: Bacterial strains, plasmids and oligonucleotides used in this study.

Name	Description	Reference
**Plasmids**		
p3×Flag-CMV14	CMV promoter, for mammalian expression, 3×Flagtag, Amp^R	Sigma
p3×Flag-CMV14-NdpA	For expression of Flag-NdpA in mammalian cells	This study
p3×Flag-CMV14-NdpA (K15A)	For expression of Flag-NdpA (K15A) in mammalian cells	This study
p3×Flag-CMV14-NdpA (R16A)	For expression of Flag-NdpA (R16A) in mammalian cells	This study
p3×Flag-CMV14-NdpA (L21E)	For expression of Flag-NdpA (L21E) in mammalian cells	This study
p3×Flag-CMV14-NdpA (L23E)	For expression of Flag-NdpA (L23E) in mammalian cells	This study
p3×Flag-CMV14-NdpA (△16-35)	For expression of Flag-NdpA (△16-35) in mammalian cells	This study
p3×Flag-CMV14-NdpA (△58-77)	For expression of Flag-NdpA (△58-77) in mammalian cells	This study
p3×Flag-CMV14-NdpA (△16-35,58-77)	For expression of Flag-NdpA (△16-35,58-77) in mammalian cells	This study
p3×Flag-CMV14-ERK2	For expression of Flag-ERK2 in mammalian cells	Laboratory
pcDNA6A	T7promoter, for mammalian expression, Myc tag, Amp^R	Invitrogen
pcDNA6A-NdpA	For expression of Myc-NdpA in mammalian cells	This study
pcDNA6A-NdpA (L21E)	For expression of NdpA (L21E) in mammalian cells	This study
pET-28a	For expression of recombinant protein His	GenScript
pET-28a-NdpA	For expression of recombinant protein His-NdpA	GenScript
pET-28a-NdpA (L21E)	For expression of recombinant protein His-NdpA (L21E)	This study
pGEX-6p-1-ERK2	For expression of recombinant proteinGST-ERK2	Laboratory
pACYC177	For *K. pneumoniae* expression, Amp^R, Kan^R	Y. Feng
pACYC177-NdpA	For expression of NdpA in *K. pneumoniae*	This study
pACYC177-NdpA (L21E)	For expression of NdpA (L21E) in *K. pneumoniae*	This study
pNF-κB-luc	Used in dual-luciferase assay for NF-κB pathway	F. Shao
pRL-TK	Used in dual-luciferase assay for NF-κB and MAPK pathways	F. Shao
pGal4-luc	Used in dual-luciferase assay for MAPK pathway	F. Shao
pFA-cJun	Used in dual-luciferase assay for MAPK pathway	F. Shao
RacL61	Used in dual-luciferase assay for MAPK pathway	F. Shao
pGal4-Elk	Used in dual-luciferase assay for MAPK pathway	F. Shao
RasV12	Used in dual-luciferase assay for MAPK pathway	F. Shao
V-Raf	Used in dual-luciferase assay for MAPK pathway	F. Shao
MEK1-ED	Used in dual-luciferase assay for MAPK pathway	F. Shao

pcDNA-HA-Ub	For expression of HA-Ub in mammalian cells	F. Shao
pcDNA-HA-Ub (K48R)	For expression of HA-Ub (K48R) in mammalian cells	F. Shao
pcDNA-HA-Ub (K63R)	For expression of HA-Ub (K63R) in mammalian cells	F. Shao
pcDNA-HA-Ub (K48 only)	For expression of HA-Ub (K48 only) in mammalian cells	L. zhang
pcDNA-HA-Ub (K63 only)	For expression of HA-Ub (K63 only) in mammalian cells	L. zhang
**Strains**		
E. coli DH5α	F$^-$φ80*lac*Z △ M15 △ (*lac*ZYA-*arg*F) U169*rec*A1*end*A1*hsd*R17 (r_K-, m_K+) *pho*A*sup*E44 λ$^-$*thi*$^-$1 *gyr*A96*rel*A1	Invitrogen
E.coli BL21 (DE3)	F$^-$*ompThsdS*$_B$ ($r_B^-m_B^-$) *gal dcm* (DE3)	Novagen
E. coli ATCC25922	Reference strain	ATCC25922
K. pneumoniae (KP)	Reference strain	NCTC5056
NdpA-KP	*K. pneumoniae* with NdpA expression	This study
NdpA(L21E)-KP	*K. pneumoniae* with NdpA (L21E) expression	This study
**Oligonucleotides (5'-3')**		
pACYC177-NdpA-F	GATCGGTTACCATTACCGTAGCAGCAGCTTTTTTTC	This study
pACYC177-NdpA-R	GATCGGTTACCTTAATTGCCGCCGGACGTCCG	This study
p3×Flag-CMV14-NdpA-F	ATAAGAATGCGGCCGCATGAGTCTGGATATCGACCAG	This study
p3×Flag-CMV14-NdpA-R	GGGGTACCGAATTGCCGCCGGACGTCCGGC	This study
pcDNA6A-NdpA-F	GGGGTACCATGAGTCTGGATATCGACCAG	This study
pcDNA6A-NdpA-R	CCGCTCGAGATTGCCGCCGGACGTCCGGC	This study
K15A mutation-F	CTGCATCAGTTAATCGCTCGTGATGAACAAAATC	This study
K15A mutation-R	GATTTTGTTCATCACGAGCGATTAACTGATGCAG	This study
R16A mutation-F	CTGCATCAGTTAATCAAAGCTGATGAACAAAATCTG	This study
R16A mutation-R	CAGATTTTGTTCATCAGCTTTGATTAACTGATGCAG	This study
L21E mutation-F	CGTGATGAACAAAATGAAGAGCTGGTGCTGCGC	This study
L21E mutation-R	GCGCAGCACCAGCTCTTCATTTTGTTCATCACG	This study
L23E mutation-F	GAACAAAATCTGGAGGAAGTGCTGCGCGAGTCAC	This study
L23E mutation-R	GTGACTCGCGCAGCACTTCCTCCAGATTTTGTTC	This study
△(16-35) mutation-F	GCCCTGCATCAGTTAATCAAAGTCGTCGAGATGATGGCTGAAC	This study
△(16-35) mutation-R	GTTCAGCCATCATCTCGACGACTTTGATTAACTGATGCAGGGC	This study
△ (58-77) mutation-F	GCAAAGCCTATGGCCTGTTCCTGGCGTTCAGCCGCGCGG	This study
△(58-77) mutation-R	CCGCGCGGCTGAACGCCAGGAACAGGCCATAGGCTTTGC	This study
